# Role of Zika Virus Envelope Protein Domain III as a Target of Human Neutralizing Antibodies

**DOI:** 10.1128/mBio.01485-19

**Published:** 2019-09-17

**Authors:** Emily N. Gallichotte, Ellen F. Young, Thomas J. Baric, Boyd L. Yount, Stefan W. Metz, Matthew C. Begley, Aravinda M. de Silva, Ralph S. Baric

**Affiliations:** a Department of Epidemiology, University of North Carolina at Chapel Hill School of Public Health, Chapel Hill, North Carolina, USA; b Department of Microbiology and Immunology, University of North Carolina at Chapel Hill School of Medicine, Chapel Hill, North Carolina, USA; University of Hong Kong

**Keywords:** Zika virus, chimeric virus, epitope, neutralizing antibodies

## Abstract

Zika virus (ZIKV) is a flavivirus that is structurally highly similar to the related viruses, dengue virus (DENV), West Nile virus, and yellow fever virus. ZIKV causes an acute infection that often results in mild symptoms but that can cause severe disease in rare instances. Following infection, individuals mount an adaptive immune response, composed of antibodies (Abs) that target the envelope (E) glycoprotein of ZIKV, which covers the surface of the virus. Groups have studied monoclonal antibodies and polyclonal immune sera isolated from individuals who recovered from natural ZIKV infections. Some of these antibodies bind to domain III of E (EDIII), but the functional importance of these antibodies is unknown. In this study, we aimed to determine if EDIII is a major target of the potent serum neutralizing antibodies present in people after ZIKV infection. By generating a chimeric virus containing ZIKV EDIII in a DENV4 virus backbone, our data show a minor role of EDIII-targeting antibodies in human polyclonal neutralization. These results reveal that while monoclonal antibody (MAb) studies are informative in identifying individual antibody epitopes, they can overestimate the importance of epitopes contained within EDIII as targets of serum neutralizing antibodies. Additionally, these results argue that the major target of human ZIKV neutralizing antibodies resides elsewhere in E; however, further studies are needed to assess the epitope specificity of the neutralizing response at the population level. Identification of the major epitopes on the envelope of ZIKV recognized by serum neutralizing antibodies is critical for understanding protective immunity following natural infection and for guiding the design and evaluation of vaccines.

## Abstract (precis)

Zika virus is a flavivirus that was recently introduced to Latin America, where it caused a massive epidemic. Individuals infected with ZIKV generate an immune response composed of antibodies which bind to the envelope (E) protein. These anti-E antibodies are critical in protecting individuals from subsequent infection. Multiple groups have found that many ZIKV antibodies bind to domain III of E (EDIII), suggesting that this region is an important target of neutralizing antibodies. Here, we generated a chimeric virus containing ZIKV EDIII in a dengue virus backbone to measure ZIKV EDIII-specific antibody responses. We found that while polyclonal ZIKV immune serum contains antibodies targeting EDIII, they constitute only a small fraction of the total population of antibodies that neutralize ZIKV. Further studies are needed to define the main targets on the viral envelope recognized by human neutralizing antibodies, which is critical for guiding the development of ZIKV vaccines.

## OBSERVATION

Zika virus (ZIKV) was isolated in Uganda in 1947 and introduced into Latin America where it caused an epidemic with millions of infections. ZIKV is genetically and antigenically similar to related flaviviruses such as dengue virus (DENV), West Nile virus (WNV), and yellow fever virus (1, 2). Decades of research into the immune response that occurs following DENV infection revealed that neutralizing antibodies (Abs) targeting the envelope protein are a critical component of protective immunity (1). Despite their protective role, antibodies are also implicated in enhancing disease in secondary infections. Because of the high degree of homology between DENV and ZIKV, there is extensive antibody cross-reactivity (both neutralizing and enhancing) (3). However, there is growing evidence that in people, prior DENV infection partially protects against subsequent ZIKV infection (4, 5). It is critical to fully define the human immune response to ZIKV natural infection to better evaluate next-generation vaccine design (1, 6).

Following ZIKV infection, individuals mount an IgG response that is predominantly directed against the envelope glycoprotein (E) (1). Multiple groups have sought to identify the epitopes targeted by human monoclonal antibodies (MAbs) against ZIKV, as they can be informative of the polyclonal antibody repertoire (3, 7–11). While MAbs have been identified that target all regions of E (domains I, II, and III), the majority of antibodies described target EDIII (3, 7–11). Additionally, multiple groups have estimated that a large fraction of polyclonal immune sera and the B-cell repertoire also target EDIII, concluding that this is therefore the primary target of ZIKV antibodies (7, 9, 11, 12). In contrast, following DENV or WNV infection, only a small fraction of antibodies target EDIII, and those that do contribute very little to total polyclonal neutralization (1, 13). Importantly, there have not been any comprehensive studies directly comparing the roles of EDIII antibodies against DENV, WNV, and ZIKV. People infected with ZIKV develop high levels of ZIKV-specific serum neutralizing antibodies, but it is unknown if EDIII is a major target of these antibodies. Using reverse genetics, we sought to develop a tool to track ZIKV EDIII-specific antibodies and to estimate their contribution to ZIKV neutralization.

**FIG 1:**
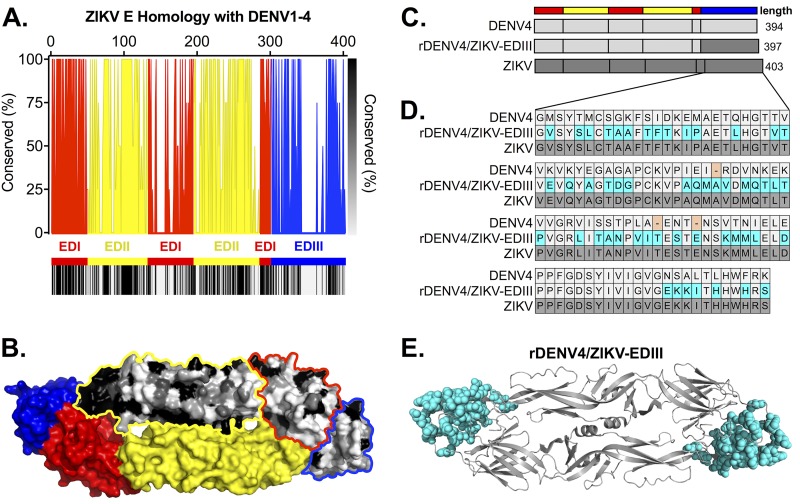
ZIKV E homology and recombinant virus design. (A) (Top) ZIKV E protein sequence homology with DENV1 to DENV4, graphed as the percentage of DENV residues that match ZIKV residues (e.g., a ZIKV residue matching two DENV serotypes = 50% conserved), color-coded by domains (with EDI, EDII, and EDIII color-coded as red, yellow, and blue, respectively). The numbers at the top of the graph correspond to amino acid position. (Bottom) The heat map displays the same ZIKV homology as displayed in the graph (black = 100% conserved, white = 0% conserved). (B) ZIKV protein dimer (PDB 5IZ7) with bottom monomer color-coded by domains and top monomer color-coded by homology to DENV as shown in panel A. (C) Design of rDENV4/ZIKV-EDIII chimeric virus. (D) EDIII amino acid alignment of DENV, ZIKV, and chimeric rDENV4/ZIKV-EDIII. Amino acids missing in DENV4 are highlighted in pink. (E) DENV protein dimer (PDB 1OAN) showing altered residues (highlighted in cyan).

Across the E ectodomain, ZIKV has high degrees of homology with DENV1 to DENV4 in EDI and EDII, which contain highly conserved regions (e.g., fusion loop) (Fig. 1A and B) (3, 12). EDIII is the least conserved, containing highly variable regions (Fig. 1A and B) (3, 12). To map ZIKV EDIII-targeting antibodies, we generated a chimeric recombinant DENV4 virus containing EDIII from ZIKV (rDENV4/ZIKV-EDIII) (Fig. 1C). The chimeric virus encodes 52 ZIKV amino acids that differ from DENV4, including the addition of three (Fig. 1D). These amino acids span EDIII and include surface-exposed as well as internally facing and cryptic residues (Fig. 1E).

**FIG 2:**
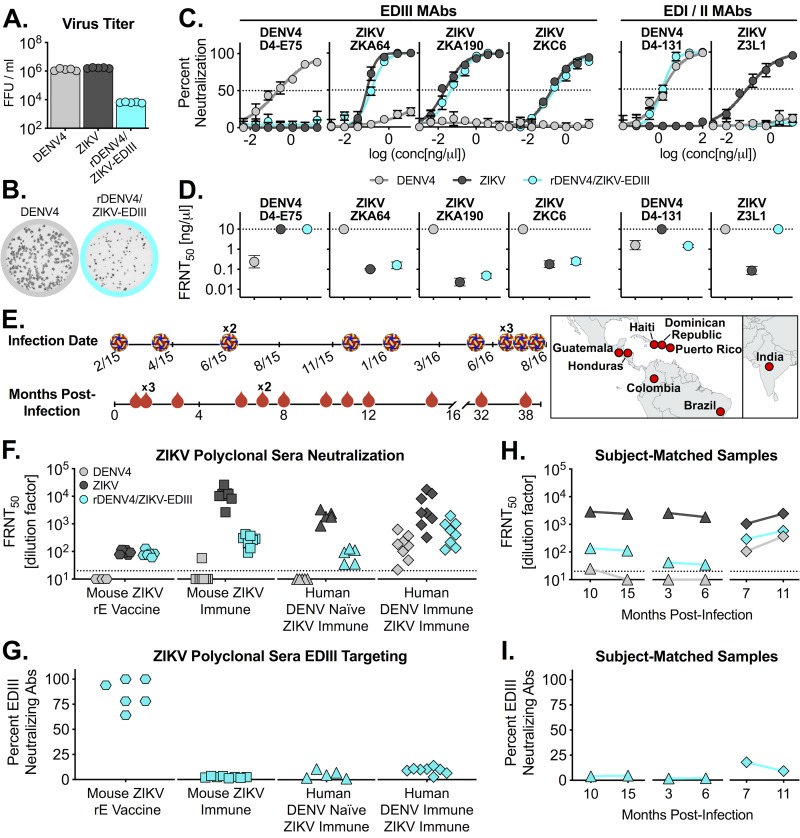
rDENV4/ZIKV-EDIII tracks with ZIKV-specific EDIII-targeting Abs. (A and B) DENV4, ZIKV, and rDENV4/ZIKV-EDIII infectious titer (A) and focus morphology (B). FFU, focus-forming units. (C) Neutralization curves of viruses by EDIII-specific and EDI/II-specific MAbs. (D) Fifty percent focus reduction neutralization titer (FRNT_50_) (representing the concentration required to neutralize 50% of virus) determined for each MAb. The dotted line represents the limit of detection (LOD). Viruses not neutralized at the highest antibody concentration are plotted at the LOD. (E) Infection date, months postinfection of samples analyzed, and location of infection for human ZIKV immune sera. (F and H) Neutralization of viruses by mouse ZIKV rE vaccine sera, mouse ZIKV immune sera, and human ZIKV immune sera from DENV-naive individuals and DENV-immune individuals (F) and subject-matched human immune sera collected at two times postinfection (H). The *y*-axis data represent FRNT_50_ values, the dotted lines represent the LOD, and viruses not neutralized are plotted at half the LOD. (G and I) Percentages of neutralizing antibodies targeting EDIII in ZIKV polyclonal sera (G) and subjected-matched samples (I) were calculated from the data presented in panels F and H as follows: (rDENV4/ZIKV-EDIII FRNT_50_ − DENV4 FRNT_50_)/(ZIKV FRNT_50_) × 100.

rDENV4/ZIKV-EDIII reached a lower titer compared to both DENV4 and ZIKV (Fig. 2A) and had smaller foci morphology relative to the parental DENV4 strain (Fig. 2B). It is possible that chimerization, in addition to attenuating the virus, altered another aspect of virus biology, such as maturation. To confirm ablation of the DENV4 EDIII epitope and transplantation of ZIKV EDIII, the viruses were evaluated for their ability to be neutralized by EDIII MAbs. rDENV4/ZIKV-EDIII was not neutralized by DENV4 EDIII-specific MAb D4-E75, whereas it was potently neutralized by three different ZIKV EDIII-specific MAbs (ZKA64, ZKA190, and ZKC6), with comparable 50% focus reduction neutralization titers (FRNT_50_) (Fig. 2C and D) (3, 14). To ensure that distal, non-EDIII epitopes were not disrupted and that their presentation was not altered, we measured neutralization by DENV4 and ZIKV EDI/II hinge antibodies D4-131 and Z3L1 (10, 15). rDENV4/ZIKV-EDIII maintained neutralization by D4-131 and did not gain neutralization to Z3L1 (Fig. 2C and D), confirming that distal epitopes were not disrupted, nor was nonspecific ZIKV neutralization gained.

We next used rDENV4/ZIKV-EDIII to measure polyclonal antibody responses in mice and humans. Human immune sera came from individuals who experienced ZIKV infection in geographically diverse locations (Central and South America, the Caribbean, and India) and from early (1 month) to late convalescent (>3 years) times postinfection (Fig. 2E). Mice vaccinated with ZIKV recombinant E (rE) generated ZIKV neutralizing antibodies that did not cross-neutralize DENV4 but efficiently neutralized rDENV4/ZIKV-EDIII, demonstrating that the majority (∼85%) targeted EDIII (Fig. 2F and G), similarly to what has previously been shown with DENV rE vaccination in mice (16). In contrast, while ZIKV-infected mice generated ZIKV-specific neutralizing antibodies, a much smaller fraction tracked with EDIII (Fig. 2F and G). Importantly, this highlights that rDENV4/ZIKV-EDIII can be used to track ZIKV-specific polyclonal antibody responses targeting EDIII.

Sera from people who experienced primary ZIKV infections (DENV-naive individuals) strongly neutralized ZIKV and weakly neutralized rDENV4/ZIKV-EDIII (Fig. 2F). Approximately 5% of ZIKV-specific neutralizing antibodies tracked with EDIII (Fig. 2G). Sera from DENV-immune individuals who were infected with ZIKV had high and intermediate levels of neutralizing antibodies to ZIKV and DENV4, respectively. In this population, only ∼9% of the ZIKV-specific neutralizing antibodies tracked with EDIII (Fig. 2F and G). For three individuals, we analyzed sera from multiple times postinfection and found that, regardless of timing, only a small fraction of ZIKV-specific neutralizing antibodies targeted EDIII, suggesting that the EDIII specificity of the polyclonal antibody response is not dynamic, nor dependent on acute versus convalescent variables (Fig. 2H and I). Together, these results revealed that across a highly diverse panel of ZIKV human immune sera, a minor role, if any, for EDIII, but further studies are needed to assess epitope specificity of the neutralizing response at the population level.

By studying the binding properties of serum antibodies in ZIKV patients to recombinant E protein or EDIII, investigators have concluded that EDIII is a major target (1). However, by using only recombinant antigens for characterizing flavivirus immune sera and MAbs, one underestimates the levels of antibodies targeting quaternary epitopes that are displayed only on intact virions. Our results suggest that EDIII-targeting antibodies account for a small fraction of the total amount of serum neutralizing antibodies following ZIKV infection as well. Although EDIII-binding antibodies are present in high levels in immune sera (11, 12), they appear to be contribute little to total neutralization, similarly to what has previously been shown for DENV (13). Additionally, some groups isolated ZIKV MAbs based on their ability to bind rEDIII, biasing their MAb repertoire to only those which have at least a majority of their epitope contained within EDIII. In contrast, by screening antibodies by binding to whole ZIKV, multiple groups have identified strongly neutralizing ZIKV-specific antibodies that target complex epitopes present only on the intact virion (3, 10, 17). It has been shown for DENV that the antibodies targeting these quaternary epitopes are primarily responsible for polyclonal neutralization, and growing evidence suggests this is the case for ZIKV as well (17). Generating chimeric viruses that recreate ZIKV quaternary epitopes would allow one to measure the contribution of these complex antibodies to total polyclonal neutralization.

Comprehensive analysis of the human immune response to ZIKV infection at the population level is critical to understanding protective immunity. Antigenic similarity between DENV and ZIKV leads to serological cross-reactivity and complicates analyses of ZIKV-specific antibody responses, especially in DENV-immune individuals (12, 18–20). Protein binding-based assays performed to distinguish DENV and ZIKV infections, while critical for accurately diagnosing infection history (12, 20), may oversimplify the complex neutralizing antibody response following infection. Therefore, additional tools (e.g., epitope transplant viruses) (21) and techniques (e.g., neutralization-based depletion assays) (17, 18) are needed to precisely map the targets of and the contributions to neutralization of different antibodies in ZIKV immune sera. This work builds on the utility of EDIII chimeric flaviviruses, which have previously been generated to map antibody responses (21) or to study aspects of pathogenesis (22). Moving forward, ZIKV vaccines must be designed to elicit responses directed to these important epitopes and evaluated based on their ability to generate antibodies targeting these critical sites (6).

## 

### Viruses.

Amino acid alignment was generated using DENV1 West Pac 74, DENV2 S16803, DENV3 UNC3001, DENV4 Sri Lanka 92, and ZIKV H/PF/2013. Viruses were generated as previously described (21, 23). Briefly, DNA encoding recombinant sequences (approved by the Institutional Biosafety Committee of the University of North Carolina at Chapel Hill [UNC] for use of recombinant virus at biosafety level 2) was introduced into a DENV4 infectious clone. Plasmid DNA was digested and ligated and T7 transcribed. Viral RNA transcripts were electroporated into C6/36 cells, and supernatant was passaged onto C6/36 cells and harvested to make working stocks.

### Cells.

C6/36 cells were grown in minimum essential medium with 5% fetal bovine serum, 100 U/ml penicillin, 100 μg/ml streptomycin, 0.25 μg/ml amphotericin-B, and nonessential amino acids at 32°C with 5% CO_2_.

### Sera.

Mouse vaccine sera come from BALB/c mice vaccinated with ZIKV recombinant E protein as described previously (24). Mouse immune sera come from ZIKV-infected C57BL/6 mice as previously described (25). Anonymized human sera were obtained from a previously described Arbovirus Traveler Collection at UNC (18), collected under Institutional Review Board approval.

### Focus reduction neutralization test.

C6/36 cells were seeded 1 day prior to infection. MAbs and sera were diluted, mixed with virus, incubated for 1 h at 32°C, and added to cells for an additional hour at 32°C. Overlay was added and incubated for 4 days. Cells were washed with phosphate-buffered saline, fixed with 50% acetone–50% methanol, blocked in milk, and stained with anti-E MAb 1M7 and horseradish peroxidase (HRP)-labeled secondary antibody. Foci were developed using TrueBlue substrate.
